# Exploring Maternal Health Care-Seeking Behavior of Married Adolescent Girls in Bangladesh: A Social-Ecological Approach

**DOI:** 10.1371/journal.pone.0169109

**Published:** 2017-01-17

**Authors:** Asm Shahabuddin, Christiana Nöstlinger, Thérèse Delvaux, Malabika Sarker, Alexandre Delamou, Azucena Bardají, Jacqueline E. W. Broerse, Vincent De Brouwere

**Affiliations:** 1 Woman and Child Health Research Centre, Department of Public Health, Institute of Tropical Medicine, Antwerp, Belgium; 2 ISGlobal, Barcelona Centre for International Health Research (CRESIB), Hospital Clínic-Universitat de Barcelona, Barcelona, Spain; 3 Athena Institute for Research on Innovation and Communication in Health and Life Sciences, VU University, Amsterdam, The Netherlands; 4 Unit of HIV/AIDS Policy, Department of Public Health, Institute of Tropical Medicine, Antwerp, Belgium; 5 James P Grant School of Public Health, BRAC University, Dhaka, Bangladesh; 6 Centre National de Formation et de Recherche en Santé Rurale de Maferinyah, Forécariah, Guinea; Centre Hospitalier Universitaire Vaudois, FRANCE

## Abstract

**Background:**

The huge proportion of child marriage contributes to high rates of pregnancies among adolescent girls in Bangladesh. Despite substantial progress in reducing maternal mortality in the last two decades, the rate of adolescent pregnancy remains high. The use of skilled maternal health services is still low in Bangladesh. Several quantitative studies described the use of skilled maternal health services among adolescent girls. So far, very little qualitative evidence exists about attitudes and practices related to maternal health. To fill this gap, we aimed at exploring maternal health care-seeking behavior of adolescent girls and their experiences related to pregnancy and delivery in Bangladesh.

**Methods and Findings:**

A prospective qualitative study was conducted among thirty married adolescent girls from three Upazilas (sub-districts) of Rangpur district. They were interviewed in two subsequent phases (2014 and 2015). To triangulate and validate the data collected from these married adolescent girls, key informant interviews (KIIs) and focus group discussions (FGDs) were conducted with different stakeholders. Data analysis was guided by the Social-Ecological Model (SEM) including four levels of factors (individual, interpersonal and family, community and social, and organizational and health systems level) which influenced the maternal health care-seeking behavior of adolescent girls. While adolescent girls showed little decision making-autonomy, interpersonal and family level factors played an important role in their use of skilled maternal health services. In addition, community and social factors and as well as organizational and health systems factors shaped adolescent girls’ maternal health care-seeking behavior.

**Conclusions:**

In order to improve the maternal health of adolescent girls, all four levels of factors of SEM should be taken into account while developing health interventions targeting adolescent girls.

## Introduction

Adolescent pregnancies are now a global concern as they are not only recognized as a risk for adolescent mothers and their newborns, but a vital development issue for any society. It is estimated that every year about 16 million girls aged between 15–19 years give birth in low-income countries (LIC) [1] and 70,000 die of complications during pregnancy and childbirth [2].

Several studies have pointed out an increased rate of adverse maternal and perinatal health conditions and outcomes associated with adolescent pregnancies, such as preterm birth, eclampsia, puerperal endometritis, systematic infections, low birth weight, perinatal death and maternal death [1,3–9]. However, other literature suggested that lower maternal age is not associated with adverse maternal health outcomes [10–12]. This conflicting evidence can be explained by variation in methodologies of studies and their context. Notwithstanding, it is evident that timely uptake of quality maternal health services is essential in decreasing the incidence of adverse maternal health outcomes among women in all age groups [13–17].

Despite substantial progress in reducing maternal mortality in the past two decades, rates of adolescent pregnancy remain high [18] and the use of skilled maternal health services is still low in Bangladesh [18]. Data from the most recent Bangladesh DHS (demographic and health survey 2014) show that only about 31% of women who had given birth three years prior to the survey received at least four antenatal care (ANC) for their most recent delivery, only 42% of the delivery were assisted by a skilled birth attendant, and that 37% of births took place at a health facility [19]. However, among adolescent girls, about 20% did not receive any ANC, while 58% of the deliveries took place at home without assistance from skilled attendants [19].

In South Asia, Bangladesh has the highest rate (35%) of adolescent pregnancy [19,20]. Although the legal age of marriage in Bangladesh for girls is 18 years, about 66% of the women get married before that age [21]. This huge proportion of child marriage contributes to the high rates of pregnancies among adolescent girls in Bangladesh. Existing literature indicates that women’s age, level of education, place of residence, decision-making autonomy, socio-cultural practices and norms, health beliefs and access and availability of quality health services influence the use of skilled maternal health services among women, including adolescents in Bangladesh [22–29].

This evidence comes predominantly from quantitative studies, and so far, very little qualitative research has been performed on the maternal health-care-seeking behavior of adolescent girls. Moreover, no qualitative study has followed up and explored the maternal health care-seeking behavior of adolescents during and after pregnancy. To fill this gap, we opted for conducting a prospective qualitative study. It aimed at exploring adolescent girls’ maternal health care-seeking behavior and their experiences related to pregnancy and delivery in Bangladesh. More specifically, we explored whether adolescent girls were able to deliver in the place they intended to and if not, which factors influenced their health care-seeking behavior. Findings of this study will serve to inform and support maternal health programs and policies targeting adolescent girls with the aim to improve their maternal health.

## Materials and Methods

### Study design

This was a prospective qualitative study in which data were collected from married adolescent girls in two phases. Multiple data sources were used to triangulate and validate the findings including in-depth interviews (IDIs) with married adolescent girls, key informant interviews (KIIs) and focus group discussions (FGDs) with different stakeholders.

### Study setting

This study was conducted in Rangpur district in Rangpur division, Bangladesh. Most recent data show that Rangpur division has the highest rate (37%) of teenage pregnancy in Bangladesh [19]. We purposively selected married adolescent girls residing in three sub-districts of Rangpur district: Mithapukur, Kaunia and Badarganj. Socio-economic conditions, cultural practices and beliefs and access to maternal health services are quite similar for the people living in these three sub-districts. Community health workers from BRAC (an international development organization based in Bangladesh) have been delivering door-to-door family planning and maternal care services in almost every village in the three sub-districts of Rangpur. In addition, LAMB [a non-governmental organization (NGO)] has been providing free ANC, postnatal care (PNC) and delivery services via its Safe Delivery Unit (SDU) at Badarganj sub-district.

### Study population

Qualitative data were collected from a wide range of respondents. In addition to married adolescent girls, the main study population, we collected data from community health workers, community people, family members of adolescent girls (mothers-in-law and husbands), representatives from the government, NGOs and health providers. Table 1 shows a list of study participants and data collection methods.

**Table 1:** Data collection methods and study respondents in Phase 1 and Phase 2.

Methods	Participants	
	First phase (December, 2014)	Second Phase (December, 2015)
**In-depth interviews (IDIs)**	Married pregnant adolescent girls (n = 25)	Same girls who were pregnant during the first phase (n = 23) [lost to follow-up, n = 2]
	Married non-pregnant adolescent girls (n = 10)	Same non-pregnant adolescent girls participated in the first phase (n = 7) [lost to follow-up, n = 3] Husbands of adolescent girls (n = 2)
**Focus group discussions (FGDs)**	FGD1: With community health workers [BRAC Shasthya Shebika (SS)] (n = 6); FGD2: With community people (n = 7) including school teachers, community leaders and religious leaders; FGD 3: With mothers-in-law who had adolescent daughters-in-law (n = 6)	
**Key informant interviews (KIIs**)	Government officer (deputy director of family planning) in Rangpur district (n = 1), NGO officials who were working on a maternal health project in Rangpur district (n = 2) and Medical doctor (gynecologist) who was working in Rangpur medical college hospital (n = 1)	

### Data collection

We collected data purposively from different types of respondents to obtain rich data. In-depth interviews (IDIs) were conducted with married pregnant and non-pregnant adolescent girls in two phases. During the first phase (December 2014), pregnant adolescents were asked about their knowledge, perception and practices related to maternal healthcare services and their intended delivery places and methods. Non-pregnant adolescents were interviewed about their knowledge, perception and practices related to family planning methods and intention of childbearing. During the second phase of the study (December 2015), the same participants were asked about their experiences during pregnancy and delivery care, whether they had become pregnant or not, and if any, what their experiences were with maternal healthcare services, such as ANC. For both groups, the information collected in the first phase was combined with that of the second phase. Four female research assistants (anthropologists, experienced in conducting IDIs and FGDs) collected data from the adolescent girls during these two phases. Research assistants were trained to conduct interviews in a way that biases were reduced (i.e. dominant respondent bias, shyness bias). KIIs were conducted with representatives of the government, NGOs, and hospital personnel who had been working in a public hospital in Rangpur district. Finally, three FGDs were conducted with community health workers, members of a village maternal health committee, and adolescents’ mothers-in-law in order to validate the data gathered via IDIs and KIIs as well as to explore common practices and barriers to the use of maternal health services.

BRAC field staff working on a maternal health project in Rangpur district supported the research team in identifying married pregnant and non-pregnant adolescent girls in the community.

The interview guides were pre-tested in Rangpur Sadar Upazila, Rangpur district and adapted. All topic guides were developed in English and translated into Bangla, before pre-testing. Due to logistical issues (e.g. time constraint, difficulties to find respondents to gather in a place) the FGD topic guide could not be piloted.

### Data analysis and theoretical framework

**Fig 1:**
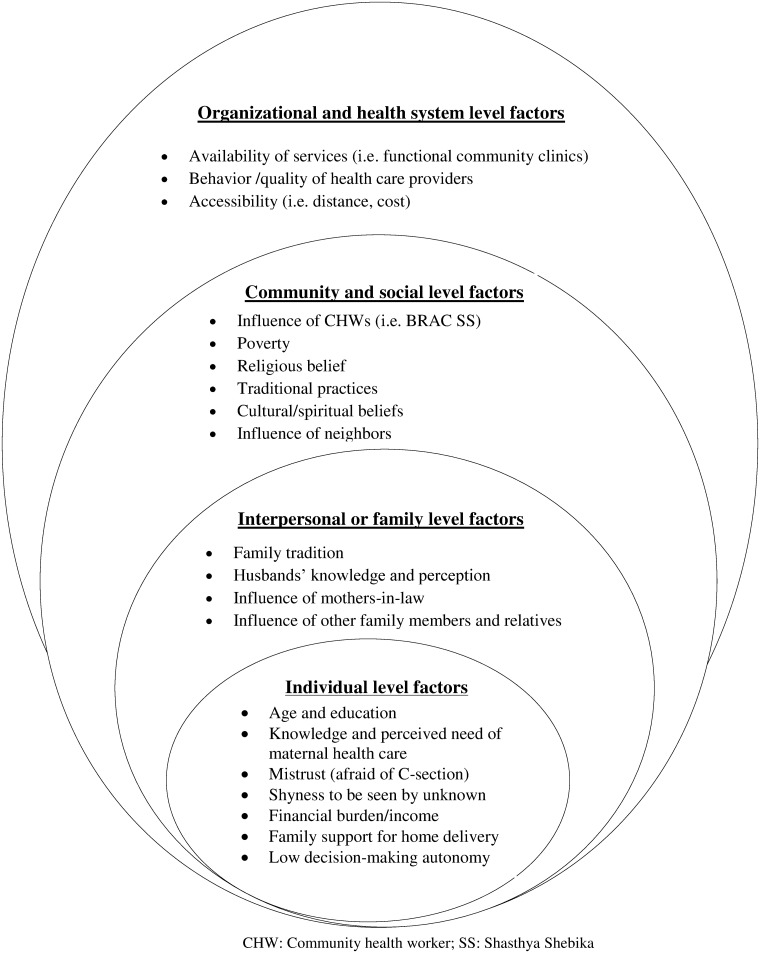
Factors influencing adolescent girls’ maternal health care-seeking behavior along the dimensions of the Social-Ecological Model.

We analyzed data with the help of MAXQDA 11 software using the Social-Ecological Model (SEM) as an initial coding guide [30]. The SEM is a theory-based framework which considers the complex interplay of multiple levels of a social system and interactions between individuals and environment within this system. The SEM thus adequately facilitated the exploration of adolescent girls’ experiences, integrating their intrapersonal, partner-related, family, community and socio-cultural contexts to produce one behavioral outcome regarding maternal health care-seeking behavior. Guided by the objectives of the study and the SEM, an initial coding framework was generated after reading a subset of the transcripts. Newly emerging text segments or codes in subsequent transcripts were inductively added to the framework to build our model of factors influencing maternal health care-seeking behavior (Fig 1). When new codes or themes were added to the framework, all data were re-scrutinized to assess their relevance. The data from IDIs, KIIs and FGDs were scrutinized several times to obtain a sense of the whole. Researchers with different backgrounds provided input to the analysis to increase its validity.

### Ethical considerations

The research protocol was approved by the Institutional Review Board (IRB) of the Institute of Tropical Medicine (ITM), Antwerp and the Ethical Review Committee (ERC) of the James P. Grant School of Public Health at BRAC University, Bangladesh. Written informed consent was obtained from all the participants. However, because of cultural issues (i.e. respondents feeling uncomfortable to sign) and participants’ illiteracy levels, from a few respondents verbal consents were obtained. Written informed consent was documented through a signature on a ‘participant information sheet and informed consent’ form and verbal informed consent was documented via audio recording. Respondents aged below 18 provided assent, while written consent was sought for them from their legal guardians/husbands. Confidentiality was strictly maintained: only the researchers had access to the data and no personally identifying information was kept that could personally identify respondents after the research had been completed.

## Results

Thirty-five in-depth interviews were conducted in total with married adolescent girls during the first phase of data collection (2014). Five girls were lost to follow-up at the second phase interviews (2015). Among the 30 adolescent girls who participated in both phases, 23 girls were pregnant during the first phase and all gave birth before to the second phase (see Table 2). Among them, one experienced a neonatal death. Of the seven initially non-pregnant girls, three became pregnant before the second phase, one gave a live birth, one experienced a neonatal death, and two did not become pregnant.

**Table 2:** Description of the adolescent girls during first and second phases of data collection.

First phase participants	Status of the participants (second phase)				Total
	Live birth	Neonatal death	Became pregnant	Not yet pregnant	
Pregnant adolescent girls (n = 23)	22	1	0	0	**23**
Non-pregnant adolescent girls (n = 7)	1	1	3	2	**7**
**Total**	**23**	**2**	**3**	**2**	**30**

### Characteristics of the respondents and use of maternal health services

Among 25 married adolescent girls, eleven delivered at a health facility while 14 delivered at home. Of those home deliveries, four were assisted by non-trained birth attendants. Among those who delivered in hospitals, seven had caesarean sections. Fourteen mothers and their newborn babies did not receive any postnatal care. ANC information was collected from 28 girls (25 had given birth and three were pregnant during the second phase of interview). Among them, nine did not receive four ANC from qualified providers (see Table 3). The majority of them received home based ANC from BRAC community health workers called Shasthya Kormi (SK). Girls from Badarganj sub-district also obtained ANC from LAMB (an NGO) Safe Delivery Unit (SDU). Among all girls, about 60% went to a private health facility at least once for an ultrasonogram mainly to know the sex of the baby.

**Table 3:** Socio-demographic characteristics and utilization of maternal health services among married adolescent girls (n = 25; n = 28 for ANC4).

Characteristics	ANC 4 (qualified providers) (n = 28)		Place of Delivery (n = 25)			Skilled birth attendants (n = 25)		Mode of Delivery (n = 25)		PNC (n = 25)	
	*Yes*	*No*	*Hospital (private)*	*Hospital (Public)*	*Home*	*Yes*	*No*	*vaginal*	*C-section*	*Yes*	*No*
**Age**											
<18 years	10	4	2	2	8	9	3	9	3	4	8
18–19 years	9	5	4	3	6	12	1	9	4	7	6
**Sub-district**											
Mithapukur	4	4	4	1	0	5	0	1	4	5	0
Badarganj	8	2	1	2	7	9	1	8	2	3	7
Kaunia	7	3	1	2	7	7	3	9	1	3	7
**Education level**											
No formal education	1	0	0	0	1	1	0	1	0	0	1
Primary school or less	6	1	0	2	5	5	2	7	0	2	5
Secondary school (any)	11	8	6	3	7	14	2	9	7	9	7
Higher than secondary	1	0	0	0	1	1	0	1	0	0	1
***Total***	***19***	***9***	***6***	***5***	***14***	***21***	***4***	***18***	***7***	***11***	***14***

Note: we considered age of girls during the second phase of data collection. Detailed socio-demographic characteristics of the study participants can be found elsewhere [31].

### Social-Ecological approach to explore maternal health care-seeking behavior

Emerging factors were grouped along the SEM layers, categorizing the study findings into individual, interpersonal and family, community and social, and organizational and health system levels factors influencing adolescents girls’ maternal health care-seeking behavior (see Fig 1).

#### Individual level factors

Among the 30 adolescent girls who took part in both phases, sixteen of them were below 18 years of age and the rest were between 18–19 years. Only one girl had completed higher than the secondary education and eight of them had primary or lower level of education. The majority of the girls had a limited knowledge on the maternal health care (ANC, hospital delivery and PNC care).

We observed that the girls’ perceived need of health care during pregnancy and childbirth influenced their use of skilled maternal health services. Most of the adolescent girls perceived pregnancy as a normal phenomenon and did not think of check-ups (i.e. ANC, PNC) or hospital delivery as necessary unless they suffered from any complication.

‘I didn’t go anywhere because I didn’t have any problem. Ruma apa (a CHW from BRAC) checked each month’

- (16 years old girl delivered at home, IDI second phase)

Almost all the girls first tried to give birth at home and when it failed, they were taken to the nearest hospital for delivery. Girls’ intention to deliver at home was because of the perceived threat of performing a C-section at the hospital and its potential consequences. During the first the interview phase one of the pregnant adolescent girls, who finally delivered her child at home, mentioned the following:

There are a lot of problems if you go to the hospital, if you do a C-section there is a problem in moving. There is a problem in eating, for three to four months you can’t do any heavy work’

-(17 years old pregnant adolescent, IDI first phase).

Three adolescent girls mentioned that they felt shy going to the hospital as there were many people at the hospital. They felt uncomfortable at the thought of being seen by male doctors in the hospital.

‘It will be better if it happens at home. It is so rush out there, there are problems and also men will be there. It is better if nurses are there to do it. I heard male doctor will be in a medical (hospital or clinic) it makes me feeling shy!’

- (18 years old girl delivered at home, IDI during the first phase)

There are a lot of people at the medical, I will be ashamed. The doctors see your body. A lot of people see your body. That’s why I didn’t feel like going to the medical. That’s why I want to deliver at home’

- (15 years old pregnant girl, IDI during the second phase)

The majority of the adolescent girls expected to have family members around them during the time of delivery. Therefore, they preferred home delivery to have direct support from the family members. Adolescent girls had little decision-making autonomy regarding maternal health care because they depended on the decisions of their husbands or parents-in-law (especially mothers-in-law) to seek maternal health care. When we asked about the uptake of ANC, one of the adolescent girls replied that:

‘He [the husband] said you have seen the doctor once so there was no need to go again. Can you see how the house is empty now, it was also empty then…Who would do the household work or take care, and then they didn’t let me go back. So I stayed home’

- (18 years old girl who experienced a neonatal death, IDI first phase)

#### Interpersonal and family level factors

Use of maternal health services was shaped by the family tradition. In most of the cases, family members demanded that pregnant girls delivered at home with the help of relatives and older women who had experience in childbirth. When a girl failed to give birth at home, she was taken to the nearby hospital. One of the adolescent girls said:

‘My pain started in the evening but I didn’t realize it. At about 12 am in the night the pain was extreme, then I realized. My husband asked me to call my mother and say that my labor pain started…I tried to push a lot but the baby was not coming out. I suffered a lot and tried till morning. At dawn they took me to the medical’

- (17 years old who recently delivered at hospital, IDI second phase)

A gynecologist who had been working for more than six years at the Rangpur Medical College Hospital confirmed such experiences in a KII:

‘I would say it (home delivery) is basically happening as a part of their tradition in the family. They do not prefer to go to a doctor or taken care by nurse as they have birth attendance and they prefer to welcome this baby within their environment’

Decisions regarding the use of ANC, skilled birth attendants at delivery and follow-up through PNC were influenced by different family members. Four types of family members were found as most influential in that aspect, i.e. husbands, parents-in-law (especially mothers-in-law), girls’ parents and senior relatives.

Husbands’ perception, beliefs and knowledge about maternal health and services availability were vital on the decisions of using skilled maternal health care for their wives. While asking about the use of PNC, one of the adolescent girls mentioned:

‘My husband said ‘let’s go to the homeopathic doctor and by the blessing of Allah the baby will be well’. Whatever my husband says turns out to be right. So we went there’

–(18 years old girl recently delivered at home, IDI second phase)

Five of the girls went to their parent’s house when pregnant and expected to deliver there, but most of them wanted to delivery at their husbands’ house. Other than husbands, mothers-in law were found to be the most influential figure in decisions related maternal healthcare. They were the first contact person for the majority of pregnant girls during pregnancy and delivery.

‘I was having pain. My mother in law realized and asked me what happened. I told her that I was having pain. My father in law went to get a rickshaw van. But before he came the baby was born at home’

-(17 years old adolescent girl recently delivered at home, IDI second phase)

This was confirmed by the KII participants. One of them also stressed the important role of mothers-in-law during pregnancy and delivery care in rural Bangladesh. Mothers-in-law also were not aware about the benefits of skilled maternal health services and their traditional perceptions on maternal health led them to not to allow their daughters-in-law to seek ANC or hospital delivery.

‘It is because mothers-in-law are the decision makers, caregiver and they believe that during the time of their pregnancies none of these things are important. Lack of awareness is the main cause for this. Our girls have no means of freedom of speech…. I would not say poverty is the main reason….. Here, they are offered to get treatment almost for free. It is observed that 98% of families do not forget to take TT (tetanus toxoid) vaccine whereas when it comes to antenatal care they seem reluctant to accept it’

-(a gynecologist from Rangpur Medical College Hospital, KII)

Participants’ parents and other older family members (i.e. sisters-in-law, grandmothers) also influenced adolescent girls’ use of maternal health services. During the time of delivery, older women such as grandmothers or aunts (husband’s aunt) often assisted in the delivery process. One of the KII participants described it as follows:

‘Every family member has the role in maternal health care but in Bangladesh, mothers-in-law have the strongest influence. Look, if a teenage girl gets married the groom’s age in an average will be 3 or 4 years older than her thus; he is also not the decision-maker and not someone who is able to guide his wife…’

(NGO official, KII)

#### Community and social level factors

We included the role of neighbors and community health workers, poverty-related factors, socio-cultural norms, traditional practices, religious and spiritual beliefs under the category of community or social factors influencing adolescent girls’ maternal health care-seeking behavior.

Most of the adolescent girls said that they received home-based ANC through BRAC’s community health worker (i.e. SK) as shown by the following quote:

‘Yes, when I was pregnant then they (BRAC’s SK) used to come and check me. Since I was 2 months pregnant health workers used to come… They used to check everything. They used to come every month’

- (17 years old girl recently delivered at public hospital, IDI first phase)

Another girl mentioned the role of LAMB community health workers. However, none of the adolescent girls mentioned community health workers deployed by the government [i.e. family welfare assistant (FWA)].

‘She (health worker) works at LAMB, she asked me to go to the clinic at Shekherhat (name of a place where the LAMB health center is) for check-ups, and I went there every month and did check-ups’

-(18 years old girl delivered at the hospital, IDI second phase).

Poverty was one of the reasons decisive for home delivery. Adolescent girls thought that going to the hospital might be expensive. Therefore, they preferred to give birth at home and to save the money for the expenses of the baby.

Religion played a very important role for home-based delivery and seeking care from religious scholar, instead of going to seek services from skilled health care providers during pregnancy and post-partum. Only two respondents were Hindus and the rest were Muslims. Muslim families did not want women to be seen by male healthcare providers.

‘They [family-in-law] are religious people. When a female patient goes to the hospital, there are male doctors checking them. That’s why he (husband) said it is better to deliver at home’

-(18 years age girl recently delivered at home, IDI second phase)

A community health worker from BRAC explained during a FGD that:

‘There are some Haji (who follow religious ritual from Macca, Saudi Arab) families who said, “We are haji people, why would some other men touch our wife?” That’s why they don’t take them to the health care center…this is bigotry’.

Cultural and spiritual beliefs aggravated the use of traditional and spiritual healers. Health problems of pregnant girls and newborns were often considered as the act of evil spirits. Such beliefs and myths perceived by adolescent girls and their family members inspired girls to seek care from traditional and spiritual healers.

‘Because my first baby died so everyone said there was an evil spirit on me. Then I got my house purified (ghor bondo kora). Me and the baby (newborn) got tabij (holly pendant) on our neck. That’s all’

- (18 years old adolescent girl recently delivered at home, IDI second phase)

When we asked about the care during pregnancy, one of the adolescent girls said that:

‘After taking the holy pedant I don’t go anymore, so I didn’t go to the doctor’

- (18 years old girl who’s baby was died, IDI second phase)

#### Organizational and health system level factors

Availability, accessibility (i.e. distance, cost) and quality of maternal health services were grouped under organizational and health systems level factors. Organization and health systems level factors also played an important role in the use of maternal health services by adolescent girls.

In terms of government health facility, the government of Bangladesh established Community Clinics (CCs) for every 6,000 population at ward/village level to provide primary health care services including ANC, vaginal delivery and PNC services almost for free particularly to the poor. However, it was found that often a community clinic was not equipped with basic instruments for ANC demotivating pregnant girls to go there for ANC check-ups. One of the community health workers from BRAC emphasized this during a FGD:

‘Sometimes BP (blood pressure) machine gets damaged in the community clinic. After convincing a pregnant woman for the check-up from the clinic, if they can’t perform the check-up due to the damage of the machine they don’t want to go there further thus they feel so irritated. They say, “You told us to go there for check-up but they didn’t do any check-up, we won’t go there anymore…”

- (BRAC SS, FGD)

Moreover, availability of services and lack of attention by the health care providers (i.e. family welfare assistant, health assistant) at the community clinic were also mentioned by a BRAC CHW during the time of FGD.

‘In some cases if we take pregnant women to the satellite clinic, sisters don’t want to perform check-ups. They say, we are in rush so come on another day…have to go to another place so, come later!’

- (BRAC SS, FGD)

Availability of health centers and accessibility were also mentioned as the reason for not seeking ANC, as illustrated by the following quote from a CHW of BRAC in a FGD:

‘Health care centers are very few. Community clinic is too far. Satellite clinic is functional for only two days in a month. If there is one more clinic day in a month, that will be better. If we combine all these, then good care will be ensured. Have to increase CSBA training’

Five girls mentioned the low quality of the service provided by public facilities as the major reason for not choosing the public hospital for childbirth.

Two adolescent girls said that they preferred private hospitals because of its nature of fast service delivery although it was costly compared to the cost of the services provided by the public hospitals. One of the FGD participants (CHWs) mentioned that:

‘It (private facility) costs. But they work instantly. In a government hospital there is too much rush. Sometimes they say, Saturday and Sunday are day off… But for some patients it is already time to deliver a baby but they don’t come…as a result baby die.’

## Discussion

This study showed that several interlinked factors influenced the use of skilled maternal health services among married adolescent girls in Bangladesh. In terms of individual level factors, adolescent girls’ knowledge and perception about pregnancy and delivery care shaped the use skilled maternal health services. During the first interview phase, almost all the adolescent girls expressed the wish to deliver at home because of their perception about the importance of maternal health care, expectation of getting family support while home delivery, being afraid of C-section and shyness to be seen by male health care providers at the hospital. However, many adolescent girls delivered at the hospital in the event of severe complications and failures of the attempt of home delivery mostly by the non-trained birth attendants.

In line with the findings from other studies, our study shows that adolescent girls often had very little decision-making autonomy. This was influenced by several intertwined factors [25,31]. A high number of child marriage in Bangladesh restricted girls from pursuing further education [32–34]. Thus, being young, low educated financially dependent on others (i.e. husbands) restricted married adolescent girls’ autonomy towards making decisions of their own health care. Furthermore, girls with low levels of education had limited knowledge about reproductive health including the importance of the skilled maternal health care, a finding which has also been corroborated in other studies [29,35]. In addition, adolescent girls often were timid to communicate with husbands and other family members because of their culturally-grounded gender-role leading to limited level of autonomy [25,31,36].

The rate of C-section reported by the study participants is alarming. Literature showed that rates of C-section increased tremendously for the past few years in Bangladesh [38]. Recent nationwide data showed that among all the deliveries about 23% were done by C-section, which is higher than WHO recommended the rate of 5% to 15% [19,39]. In particular private hospitals seemed to have encouraged C-section as a business strategy, which instilled fear of hospital delivery in pregnant girls. In the context of rural Bangladesh, married women are meant to do all the household works. Therefore, married women feared the event of C-section, as it took several weeks of recovery and they could not perform their compelling household chores.

Pregnancy and delivery care were meant to be done within family set-ups in Bangladesh societies [40,41]. Traditionally, women give birth at home without any problem which compelling adolescent girls’ to rely on past events and feel safe while giving birth within the family environment. Thus, family tradition emerged as the strongest influencing factor. Husbands and to greater extent mothers-in-law played an important role in adolescent girls’ maternal health care seeking behavior, further limiting their decision-making autonomy. In the rural context of Bangladesh, men usually are the head of a family and take most household decisions [41,42]. When it comes to the maternal health care, it is considered to be dealt with women, and mothers-in-law usually are the first person to take decisions about that. Despite this fact, it is also a common practice in Bangladesh that many girls give birth at their own parents’ house. Girls felt comfortable and expect to be taken good care while giving birth at parents’ house rather than at their in-laws’ house. In that case, girls own parents’ knowledge and perception influence maternal health care.

Under the community and social level factors, religious beliefs emerged strongly as influencing factors, as illustrated in other studies in Bangladesh [43]. Muslims constitute 90% of the population in Bangladesh, and firm believers often consider women being seen by men other than their husbands and close relatives a sin [44]. Therefore, very religious people may want pregnant woman to deliver a child within family environment and influenced for home delivery. Several studies showed that some birth preparedness practices and beliefs are linked with Bengali tradition and culture handed over from generation to generation [35]. Placement of pregnant girls in a separate room during the time of delivery for up to 40 days after delivery and filling room with smokes to make it warm are among such cultural practices [40,41]. Some beliefs like fear of evil spirits and their effects on the pregnant women are grounded in cultural beliefs rooted in rural society. Lower level of education among rural women may contribute to the major reason for maintaining such kind beliefs and practices.

The contribution of community health workers in improving Bangladesh maternal healthcare is crucial [44,45]. The presence of community health workers from BRAC and other NGO (LAMB) was noticeable in the study areas which positively influenced girls’ to use skilled maternal health services. Community health workers (i.e. HA or FWA) deployed by the government were rarely seen in the community and had less contact with pregnant girls. Despite the presence of CHWs, strong family tradition and individual and family members’ perception of maternal health care still contributed to limiting pregnant girls’ use of skilled maternal health services. Quality of health care services and the attitudes of the health personnel were mentioned as a barrier to using maternal health care and coincided with findings from other studies [46,47]. Less attention by the health care workers and non-functioning community clinics led pregnant girls’ to restrict their visit to the community clinic for ANC and PNC. The government of Bangladesh should take steps in order to strengthen the monitoring system to track the performance of the community clinics and to make all the community clinics functional.

### Strengths and limitations of the study

This study’s main strengths are its prospective study design using follow-up data from a 12 months period, and it’s comprehensive view of maternal health care-seeking behavior of married adolescent girls guided by the SEM. Data were collected from the three sub-districts of Rangpur district, therefore a study limitation is that the findings cannot be generalized to other contexts (i.e. urban setting). This is usually the case with qualitative data, which serve to explore issues in a given context and contribute to deeper insights into specific phenomena. However, we believe that the information gathered from a diverse group of respondents was rich enough to present a realistic scenario of maternal health care-seeking behavior of adolescent girls.

## Conclusions

This study revealed that all four levels of factors in the Social-Ecological Model shaped the maternal health care-seeking behavior of married adolescent girls in Bangladesh, with influences also cutting across the levels as shown for low decision-making autonomy for instance. Since adolescent girls have less decision-making autonomy and knowledge about maternal health care, interpersonal and family level factors such husbands and mothers-in-law played important roles in their utilization of skilled maternal health services. Our data show that ensuring availability of quality maternal health services in government facilities is important in building trust among young rural women. The contribution of community health workers, mainly from the private sector (e.g. BRAC) to maternal healthcare was remarkable given that pregnant adolescent girls in Bangladesh are rarely in contact with government community health workers unless girls visit CC. In order to promote the use of skilled maternal health services, interventions targeting all four levels of the SEM are needed, calling for different types of strategies addressing the different levels [48]. However, interpersonal and family levels factors should be given special attention when targeting adolescent girls, as family members are the ultimate decision makers for them and likely the most influential in the use of skilled maternal health services. ***Wider societal actors such as religious and community leaders and health services providers (i*.*e*. *CHWs) can play a vital role in sensitizing married adolescent girls’ family members (i*.*e*. *mothers-in-law*, *husbands) by informing them about the consequences of early pregnancy and the importance of using skilled maternal health services*.**
